# A New Prognostic Score for Elderly Patients with Diffuse Large B-Cell Lymphoma Treated with R-CHOP: The Prognostic Role of Blood Monocyte and Lymphocyte Counts Is Absent

**DOI:** 10.1371/journal.pone.0102594

**Published:** 2014-07-24

**Authors:** Vít Procházka, Robert Pytlík, Andrea Janíková, David Belada, David Šálek, Tomáš Papajík, Vít Campr, Tomáš Fürst, Jana Furstova, Marek Trněný

**Affiliations:** 1 Department of Hemato-Oncology, Faculty of Medicine and Dentistry, Palacký University, Olomouc, Czech Republic; 2 First Internal Department, Charles University General Hospital, Prague, Czech Republic; 3 Department of Internal Medicine-Hematooncology, University Hospital Brno, and Faculty of Medicine, Masaryk University, Brno, Czech Republic; 4 Second Department of Medicine, Department of Hematology, University Hospital and Faculty of Medicine, Hradec Králové, Czech Republic; 5 Department of Pathology and Molecular Medicine, Charles University, and Second Medical School and Faculty Hospital in Motol, Prague, Czech Republic; 6 Department of Mathematical Analysis and Applications of Mathematics, Faculty of Science, Palacký University, Olomouc, Czech Republic; West Virginia University School of Medicine, United States of America

## Abstract

**Background:**

Absolute lymphocyte count (ALC) and absolute monocyte count (AMC) have been documented as independent predictors of survival in patients with newly diagnosed Diffuse Large B-cell Lymphoma (DLBCL). Analysis of the prognostic impact of ALC and AMC in the context of International Prognostic Index (IPI) and other significant variables in elderly population treated in the R-CHOP regime has not been carried out yet.

**Methodology/Principal Findings:**

In this retrospective study, a cohort of 443 newly diagnosed DLBCL patients with age ≥60 was analyzed. All patients were treated with the R-CHOP therapy. An extensive statistical analysis was performed to identify risk factors of 3-year overall survival (OS). In multivariate analysis, only three predictors proved significant: Eastern Cooperative Oncology Group performance status (ECOG), age and bulky disease presence. These predictors were dichotomized (ECOG ≥1, age ≥70, bulk ≥7.5) to create a novel four-level score. This score predicted 3-year OS of 94.0%, 77.4%, 62.7% and 35.4% in the low-, low-intermediate, high-intermediate and high-risk groups, respectively (P<0.001). Further, a three-level score was tested which stratifies the population better (3-year OS: 91.9%, 67.2%, 36.2% in the low, intermediate and high-risk groups, respectively) but is more difficult to interpret. Both the 3- and 4-level scores were compared to standard scoring systems and, in our population, were shown to be superior in terms of patients risk stratification with respect to 3-year OS prediction. The results were successfully validated on an independent cohort of 162 patients of similar group characteristics.

**Conclusions:**

The prognostic role of baseline ALC, AMC or their ratio (LMR) was not confirmed in the multivariate context in elderly population with DLBCL treated with R-CHOP. The newly proposed age-specific index stratifies the elderly population into risk groups more precisely than the conventional IPI and its existing variants.

## Introduction

Diffuse large B-cell lymphoma (DLBCL) is one of the most frequent subtypes of lymphoma of the Western Hemisphere [1]. The median age at diagnosis is about 65 years and the majority of patients are sixty or older. Novel treatment with rituximab-containing regimens and better supportive care markedly improved the outcomes in elderly patients [2]–[4]. The improved prognosis of DLBCL in elderly patients may also be related to intrinsic biological features of the tumor [5]. In addition to clinical conditions related to age, the role of the conventional prognostic variables, included in the International Prognostic Index (IPI) [6] or novel revised IPI (R-IPI) [7], may be altered in this population. The IPI was postulated in the pre-rituximab era and some retrospective analyses show its limited predictive value: Despite being a four-level score, the IPI usually identifies only two risk subgroups. Analyses published by Ziepert et al. [8] confirm IPI as a valid predictor when analyzing data from prospective trials with rituximab-based regimens. A subanalysis of older patient population (the RICOVER-60 study) [9] showed overlaps between the high-intermediate and high IPI categories. Moreover, two of the IPI variables (ECOG, and Ann Arbor stage) did not reach statistical significance in the Cox regression model for progression-free survival (PFS) and overall survival (OS). The novel “recalculated” R-IPI is a more powerful tool for the whole population, however with a limited information value for patients older than sixty years. No patients over sixty are considered low risk due to their age. This fact, together with an increasing proportion of elderly patients in good physical conditions, advocates for age-specific prognostic tools. Advani et al. [10] published an analysis of patients older than 60 treated with R-CHOP in US intergroup studies. Their elderly IPI (E-IPI) considered age over 70 as a negative prognostic marker, and it showed a superior discrimination power compared to IPI and age-adjusted IPI (AA-IPI) [6] scores. Unfortunately, no extensive multivariate analysis of predictor variables was done. Prognostic stratification in older population should be more focused on the real “biological” age of patients and on primary variables that reflect tumor aggressiveness and immune interaction between the tumor and host. There is growing evidence of a strong predictive role of the absolute lymphocyte count (ALC), absolute monocyte count (AMC) or their ratio (lymphocyte to monocyte ratio, LMR). This supports the hypothesis that host innate immunity is critical in tumor growth control and it is a limiting factor for the efficacy of immunochemotherapy in patients with DLBCL [11]–[13]. The optimal cut-off levels of ALC and AMC may be different in various populations [14]–[15]. This fact should be taken into account when designing new ALC/AMC-based prognostic schemes [16]–[18].

This retrospective study analyzes the role of conventional clinical and laboratory parameters in an unselected cohort of elderly patients with DLBCL treated in the Czech Republic with rituximab-based chemotherapy. The original focus was on modifying the IPI score for elderly population, by incorporating the prognostic roles of AMC, ALC, and LMR. However, no prognostic role of baseline ALC, AMC or their ratio (LMR) was found in the multivariate context in elderly population with DLBCL treated with R-CHOP. On the other hand, two variants of a novel prognostic score were postulated for this population. The scores are based on age, performance status according to WHO (ECOG), and the presence of bulky disease. Both the novel scores are found to be superior to previously published schemes. The novel scores were successfully validated on an independent cohort of similar group characteristics.

## Materials and Methods

### Ethics Statement

The study was performed in accordance with the 2008 revision of the Declaration of Helsinki. All patients provided an informed written consent to anonymous processing of data on their disease. The study was approved by the ethical committee of the Faculty Hospital in Prague.

### Subjects

The Czech Lymphoma Study Group (CLSG) is a national scientific organization which provides a platform for cooperation among Czech hematologists, oncologists and hematopathologists. The Lymphoma Registry (LR) is a prospective online database founded and operated by the CLSG which collects data from newly diagnosed lymphoma patients since 2000. The CLSG database covers up to 68% of all newly diagnosed lymphoma cases [19]. It currently contains 11,122 patients with lymphoma, including 627 DLBCL patients sixty years and older treated in the rituximab era. A cohort was selected to include all patients with a histologically confirmed diagnosis of DLBCL who were sixty years or older at the time of diagnosis and were treated with the R-CHOP regime [20]. The cohort included all patients with newly diagnosed DLBCL recorded in LR between April 2002 and May 2010, to allow for at least three-year follow-up. Patients with central nervous system involvement were excluded from the study. All biopsies were reviewed by a reference hematopathologist and the final diagnosis was provided in compliance with the published World Health Organization (WHO) classification. A central review of all final diagnosis reports was carried out [21]. The cohort consists of 443 patients (clinical data summarized in Table 1). On this cohort, all univariate and multivariate statistical analyses (see below) were performed. Before constructing the predictive score, further 64 patients were excluded because of missing data (i.e. at least one of the predictors used in the final score was missing). Consequently, the comparison with existing scores and assessment of the score performance was done on a group of 379 patients. In the original CLSG query, only patients with complete data on ALC and AMC were selected. However, no prognostic role of these predictors was found (see Results). This enabled us to repeat the query without this constraint and thereby obtain a validation cohort of 162 patients from the same population. The validation cohort was selected about 1 year later than the original one.

**Table 1:** Summary of all the prognostic factors.

	OS univariate analysis		Descriptive statistics		
Prognostic factor	HR (95% CI)	P-value	Min–Max	Median	N (%)
Age [years]	1.07 (1.04, 1.10)	<0.0001	60–88	70	
ALC [×10^9^ /l]	0.67 (0.54, 0.84)	0.0006	0.01–16.64	1.41	
AMC [×10^9^ /l]	1.16 (0.83, 1.63)	0.3740	0.02–5.04	0.60	
LMR [–]	0.89 (0.81, 0.98)	0.0146	0.03–81.00	2.43	
Hemoglobine [g/l]	0.69 (0.52, 0.92)	0.0123	15–171	126	
No. of extranodal regions	1.20 (1.01, 1.43)	0.0357	0–5	1	
ECOG score	1.66 (1.41, 1.96)	<0.0001	0–4	1	
Ann Arbor stage	1.40 (1.20, 1.64)	<0.0001	1–4	3	
Sex (male)	1.32 (0.94, 1.84)	0.1050			186 (49.1)
Bulky disease (≥7.5 cm)	2.22 (1.55, 3.17)	<0.0001			136 (35.9)
Systemic symptoms present	2.44 (1.75, 3.41)	<0.0001			138 (37.1)
Bone marrow affected	1.69 (1.08, 2.66)	0.0228			49 (18.7)
LDH (≥ limit)	2.29 (1.56, 3.36)	<0.0001			226 (60.4)
B2M (≥ limit)	2.29 (1.47, 3.55)	0.0002			165 (56.5)

*Results of the univariate 3-year overall survival (OS) analyses: hazard rate (HR) with its 95% confidence interval (CI) and P-value based on the Cox regression model. Descriptive statistics of all the prognostic factors.*

### Data

The following dichotomous predictors were considered for each subject at the time of the diagnosis: sex, bulky disease presence (limit 7.5 cm) [9], bone marrow affected, presence of systemic symptoms, lactate dehydrogenase level exceeding upper limit (LDH), beta-2-microglobulin level exceeding upper limit (B2M). The following categorical predictors were considered: number of extranodal regions affected, performance status according to WHO/ECOG (0–4), and Ann Arbor stage (1–4). The following continuous predictors were considered: age, absolute lymphocyte count (ALC, ×10^9^/l), absolute monocyte count (AMC, ×10^9^/l), lymphocyte to monocyte ratio (LMR = ALC/AMC), hemoglobin level (g/l).

### Follow-up

OS was defined as the time from diagnosis of DLBCL to death from any cause. PFS was defined as time from diagnosis to lymphoma relapse, progression or death of any cause. Analyses were fitted to detect differences in survival times after 3 years of follow-up. All living patients’ OS and PFS were censored three years from the diagnosis. This was done because the prognostic factors allow for the best discrimination of the population at around three years from the diagnosis. In later years, DLBCL unrelated factors may start outweighing the DLBCL-related ones in the OS and PFS.

### Statistical methods

First, univariate analysis was performed to find out which of the risk factors are significant independent predictors of the 3-year OS. The Cox proportional hazards model was used. All independently significant predictors were consequently used in multivariate Cox regression analysis. By stepwise elimination, the least significant predictors were excluded to arrive at the final model. Only non-significant predictors were excluded. The predictors included in the final model were further dichotomized to allow for the construction of a simple predictive score (see Results). Performance of the newly proposed score was compared to existing predictive scores by means of the concordance measure and Akaike’s information criterion (AIC). Concordance measures the probability of agreement for any pair of patients, where agreement means that the patient with the shorter survival time also has the larger risk score. Comparison of survival times was performed by the Kaplan-Meier survival curve plots and log-rank tests. All statistical analyses were performed using the R software [22]. The significance level of all tests was set to 0.05. Validation was performed by means of the concordance measure and by comparing the proportional hazards of the respective risk groups in the training and the validation cohorts.

## Results

### Treatment response and survival analysis

**Figure 1:**
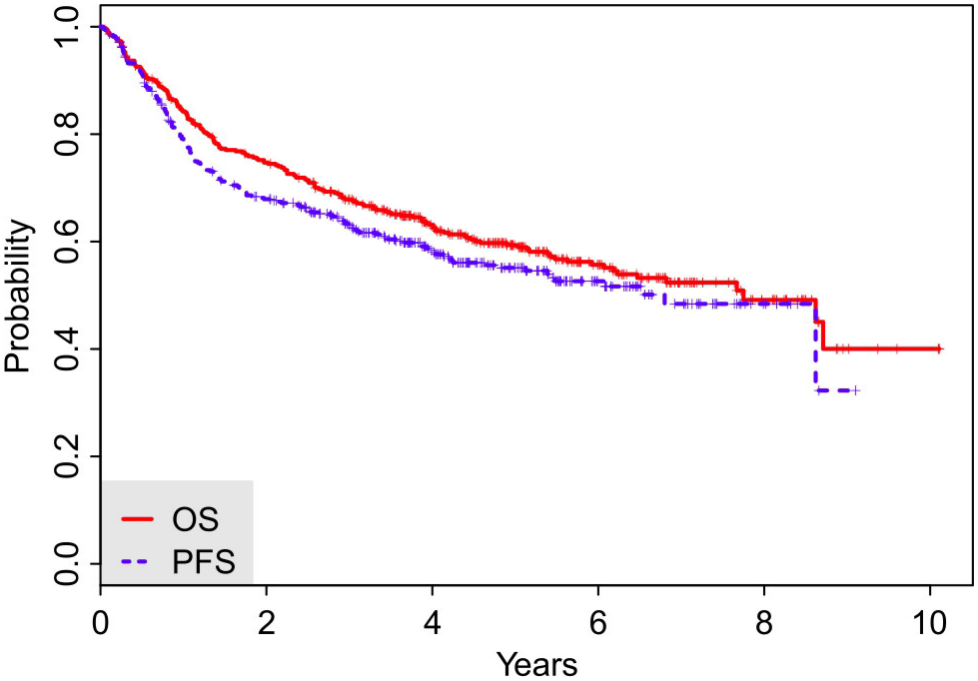
Overall survival (OS) and Progression-free survival (PFS) curves for the entire cohort. Complete follow-up.

Treatment response was available in 400 out of the 443 patients (90.3%) in the training cohort. Complete response (CR), partial response (PR), stable (SD) and progressive disease (PD) were observed in 326 (81.5%), 42 (10.5%), 3 (0.8%) and 29 (7.3%) of the patients, respectively. During the follow-up (a median of 5.06 years for the surviving patients), 188 patients died (42.4%). The 3-year OS was 67.9% (95% CI: 0.64–0.72) and the median OS was 7.8 years (95% CI: 6.2–8.7 years). 3-year PFS reached 61.1% (95% CI: 0.56–0.66), median PFS was 5.4 years (95% CI: 4.1–6.6 years), see Figure 1.

### Regression analysis

Univariate Cox regression analysis was performed on all prognostic factors listed in Table 1. The regression analysis revealed that all the considered risk factors are significant individual predictors of 3-year OS, except for the sex and AMC. In the multivariate analysis, we started by including all significant univariate predictors in a Cox proportional hazards model, and then gradually eliminated the insignificant ones. The final model contains only age, bulky disease presence (dichotomous) and ECOG performance status (0–4) (see Table 2). The AMC has no prognostic impact and the significance of ALC disappeared in the multivariate context.

**Table 2:** Multivariate model results.

	OS multivariate analysis	
Prognostic factor	HR (95% CI)	P-value
Age [years]	1.08 (1.04, 1.11)	<0.0001
Bulky disease (≥7.5 cm)	1.76 (1.21, 2.57)	0.0033
ECOG score (0–4)	1.61 (1.33, 1.95)	<0.0001

*Results of the final multivariate 3-year overall survival (OS) model: Hazard rate (HR) with its 95% confidence interval (CI) and P-value based on the Cox regression model.*

### Predictive score construction

**Figure 2:**
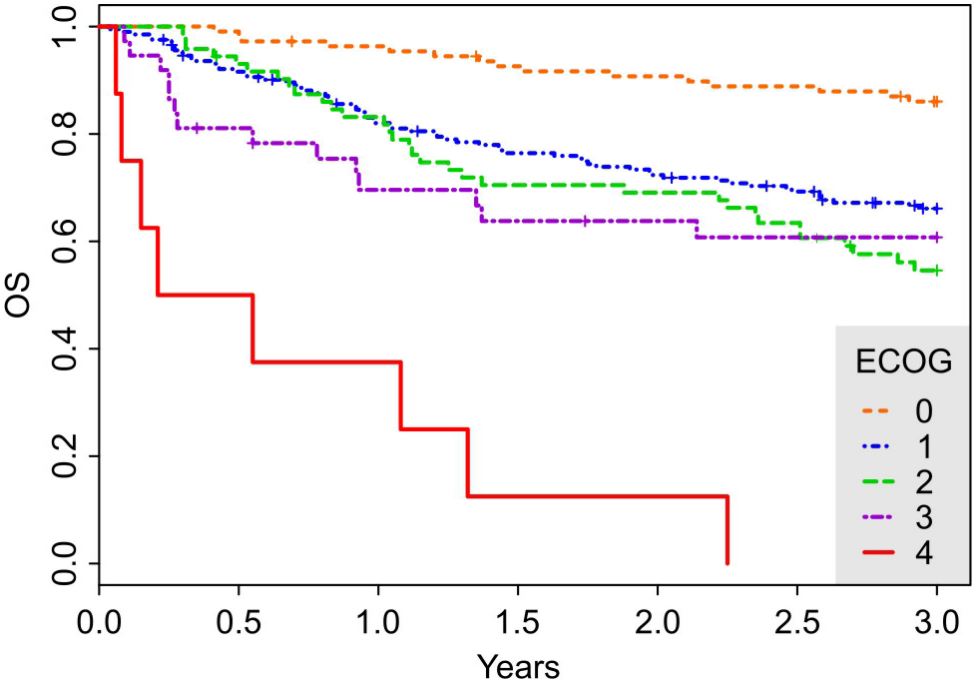
3-year overall survival (OS) curves for the entire cohort stratified by the ECOG score. The hazard rates of groups 1, 2, 3, 4 relative to group ECOG = 0 are 2.85, 3.89, 3.82, 24.58, respectively. Notice the clear separation of the group ECOG = 4 from the rest of the population. However, group ECOG = 4 contains only 8 patients. Optimal stratification of the population into two groups thus separates group ECOG = 0 (109 patients) from the rest of the population (334 patients).

The construction of a simple predictive score was based on the multivariate analysis results. In order to construct a simple score out of the three significant multivariate predictors, it is necessary to further discretize age and ECOG. Otherwise, the score would stratify the population into too many groups. Thus, we propose the following scheme: A patient gets one point for having age ≥70, one point for having bulky disease (bulk ≥7.5 cm) and one point for having ECOG ≥1. The cut-off level for age (70 years) was determined so that the hazard rate of the high risk group (age ≥70) relative to the low risk group (age below 70) is comparable to the hazard rates of the two remaining predictors. Moreover, the median age of the cohort is 70 years, and the E-IPI prognostic score [10] uses the same cut-off. The ECOG score was discretized according to Figure 2 which clearly differentiates patients with ECOG = 0 from the rest of the population. The three dichotomized predictors remain significant: the hazard rates (HR) for age ≥70, bulky disease and ECOG ≥1 are 2.20 (95% CI: 1.52–3.19, P<0.0001), 2.00 (95% CI: 1.39–2.87, P = 0.0002) and 3.18 (95% CI: 1.70–5.95, P = 0.0003), respectively.

Presence or absence of these three binary predictors (risk factors) stratifies the entire population into eight groups (see Table 3). It is convenient to define a four-level prognostic score (here denoted as ABE4-Score to remember that it is derived from Age, Bulk, and ECOG), analogously to IPI, as the number of risk factors present in the patient. Thus, patients without any risk factor (‘Group I’) are assigned ABE4-Score = 0 (N = 51) and represent the low risk group, patients with 1 risk factor (‘Groups ii–iv’) are assigned ABE4-Score = I (N = 125) and represent the low-intermediate risk group, patients with 2 risk factors (‘Groups v–vii’) are assigned ABE4-Score = II (N = 149) and represent the high-intermediate risk group, and patients with all three risk factors (‘Group viii’) are assigned ABE4-Score = III (N = 54) and represents the high risk group. Table 4 shows the hazard rates of the individual ABE4-Score groups calculated by means of the Cox proportional hazards model with the ABE4-Score as the only predictor.

**Table 3:** Construction of the ABE4-Score.

		Risk factors present					
Group	ABE4-Score	Age ≥70	Bulk ≥7.5	ECOG ≥1	N (%)	HR	P-value
I	0	0	0	0	51 (13.5)		
Ii	I	1	0	0	22 (5.8)	1.52	0.6444
iii		0	1	0	14 (3.7)	2.56	0.3028
Iv		0	0	1	89 (23.5)	5.17	0.0073
V	II	1	1	0	7 (1.8)	14.77	0.0004
vi		1	0	1	81 (21.4)	8.17	0.0005
vii		0	1	1	61 (16.1)	6.61	0.0023
viii	III	1	1	1	54 (14.2)	18.90	<0.0001

*Presence (1) or absence (0) of the three risk factors stratifies the population into eight groups (Group i–viii). The ABE4-Score is defined as the number of risk factors present in the patient. Results of the univariate Cox regression analysis: hazard rate (HR) and P-value with reference group being Group i (ABE4-Score = 0).*

**Table 4:** Summary of the scoring systems.

Group		N (%)	Estimated 3-year OS [%] (95% CI)	HR (95% CI)	P-value
**Four-level scores**					
ABE4					
	Low (0)	51 (13.5)	94 (88, 100)		
	Low-intermediate (I)	125 (33.0)	77 (70, 85)	4.15 (1.26, 13.66)	0.0191
	High-intermediate (II)	149 (39.3)	63 (55, 71)	7.75 (2.42, 24.78)	0.0006
	High (III)	54 (14.2)	35 (24, 52)	18.86 (5.78, 61.58)	<0.0001
IPI					
	Low	90 (24.1)	83 (76, 91)		
	Low-intermediate	93 (24.9)	75 (66, 84)	1.58 (0.83, 3.03)	0.1663
	High-intermediate	98 (26.2)	65 (56, 75)	2.37 (1.29, 4.36)	0.0053
	High	93 (24.9)	51 (41, 62)	3.86 (2.14, 6.93)	<0.0001
Age-adjusted IPI					
	Low	83 (22.3)	82 (74, 91)		
	Low-intermediate	117 (31.5)	77 (70, 85)	1.29 (0.68, 2.43)	0.4357
	High-intermediate	106 (28.5)	64 (56, 74)	2.19 (1.20, 3.99)	0.0106
	High	66 (17.7)	40 (29, 54)	4.80 (2.64, 8.73)	<0.0001
Elderly IPI					
	Low	142 (38.2)	82 (76, 89)		
	Low-intermediate	80 (21.5)	72 (63, 83)	1.78 (1.01, 3.16)	0.0484
	High-intermediate	91 (24.4)	59 (50, 70)	2.64 (1.59, 4.40)	0.0002
	High	59 (15.9)	41 (30, 56)	5.23 (3.10, 8.81)	<0.0001
**Three-level scores**					
ABE3					
	Low	87 (23.0)	92 (86, 98)		
	Intermediate	231 (61.0)	67 (61, 74)	4.75 (2.19, 10.30)	<0.0001
	High	61 (16.0)	36 (26, 51)	13.33 (5.93, 29.95)	<0.0001
RIPI					
	Very good	5 (1.3)	80 (51, 100)		
	Good	178 (47.6)	79 (73, 85)	1.01 (0.14, 7.35)	0.9930
	Poor	191 (51.1)	58 (51, 65)	2.37 (0.33, 17.06)	0.3900
ALC/RIPI					
	Low	164 (43.9)	80 (74, 87)		
	Intermediate	141 (37.7)	63 (56, 72)	2.12 (1.36, 3.30)	0.0009
	High	69 (18.4)	50 (39, 64)	3.14 (1.93, 5.12)	<0.0001

*Distribution and outcome of patients according to the compared risk scoring systems. Results of the univariate 3-year overall survival analysis: estimated 3-year overall survival (OS) with its 95% confidence interval (CI), hazard rate (HR) with its 95% CI and P-value based on the Cox regression model. Reference group in all regression models is the lowest risk group.*

This prognostic score is easy to interpret (it represents the number of risk factors present), however, it is interesting to note that ‘Group v’ has significantly worse 3-year OS (HR with respect to ‘Group I’ is 14.8, P = 0.0004) than ‘Group vi’ (HR = 8.1, P = 0.0005) and ‘Group vii’ (HR = 6.6, P = 0.0022). The HR of ‘Group v’ is even comparable to the HR of the worst prognosis ‘Group viii’ (HR = 18.9, P<0.0001). Thus, it seems that the combination of age ≥70 and bulky disease, despite ECOG = 0, has comparably pessimistic prognosis as the group where all the risk factors are present. This suggests defining another prognostic score, this time a three-level one: according to the results of the Cox regression analysis (see Table 3), there is no significant difference in HR of Groups i, ii and iii. Thus, Groups i, ii and iii are pooled into the low risk group and are assigned ABE3-Score = 0 (N = 87), Groups iv, vi, and vii represent the intermediate risk group and are assigned ABE3-Score = I (N = 231), and Groups v and viii are assigned ABE3-Score = II (N = 61) and represent the high risk group of patients (see Table 5). The Cox proportional hazards model provides the following results with respect to the ABE3-Score low risk group (see Table 4): HR of ABE3-Score intermediate risk group is 4.75 (P<0.0001), HR of ABE3-Score high risk group is 13.33 (P<0.0001). The estimated 3-year OS with ABE3-Score stratification is: 0.92 (95% CI: 0.86–0.98) for ABE3-Score low risk group, 0.67 (95% CI: 0.61–0.74) for ABE3-Score intermediate risk group and 0.36 (95% CI: 0.26–0.51) for ABE3-Score high risk group. The ABE3-Score model stratifies the cohort in a more reasonable way (see the ‘Comparison’ section), however, the group sizes are not well balanced, and it is not as easily interpreted as the 4-category ABE4-Score model.

**Table 5:** Construction of the ABE3-Score.

		Risk factors present		
Group	ABE3-Score	Age ≥70	Bulk ≥7.5	ECOG ≥1
i	0	0	0	0
ii		1	0	0
iii		0	1	0
iv	I	0	0	1
vi		1	0	1
vii		0	1	1
v	II	1	1	0
viii		1	1	1

*Presence (1) or absence (0) of the three risk factors stratifies the population into eight groups (Group i–viii). The ABE3-Score pools certain groups to stratify the population into three risk groups. Note that the Groups i–viii (the first column) do not appear in ascending order in contrast to* *Table 3* *.*

### Comparison to existing scoring systems

**Figure 3:**
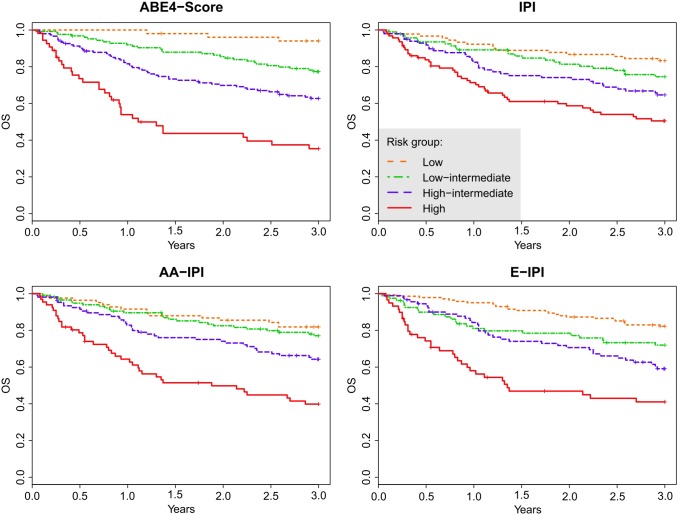
Overall survival (OS) curves for the entire cohort stratified by the 4-level scoring systems: the proposed novel ABE4-Score, and the classical IPI, AA-IPI, and E-IPI scores.

**Figure 4:**
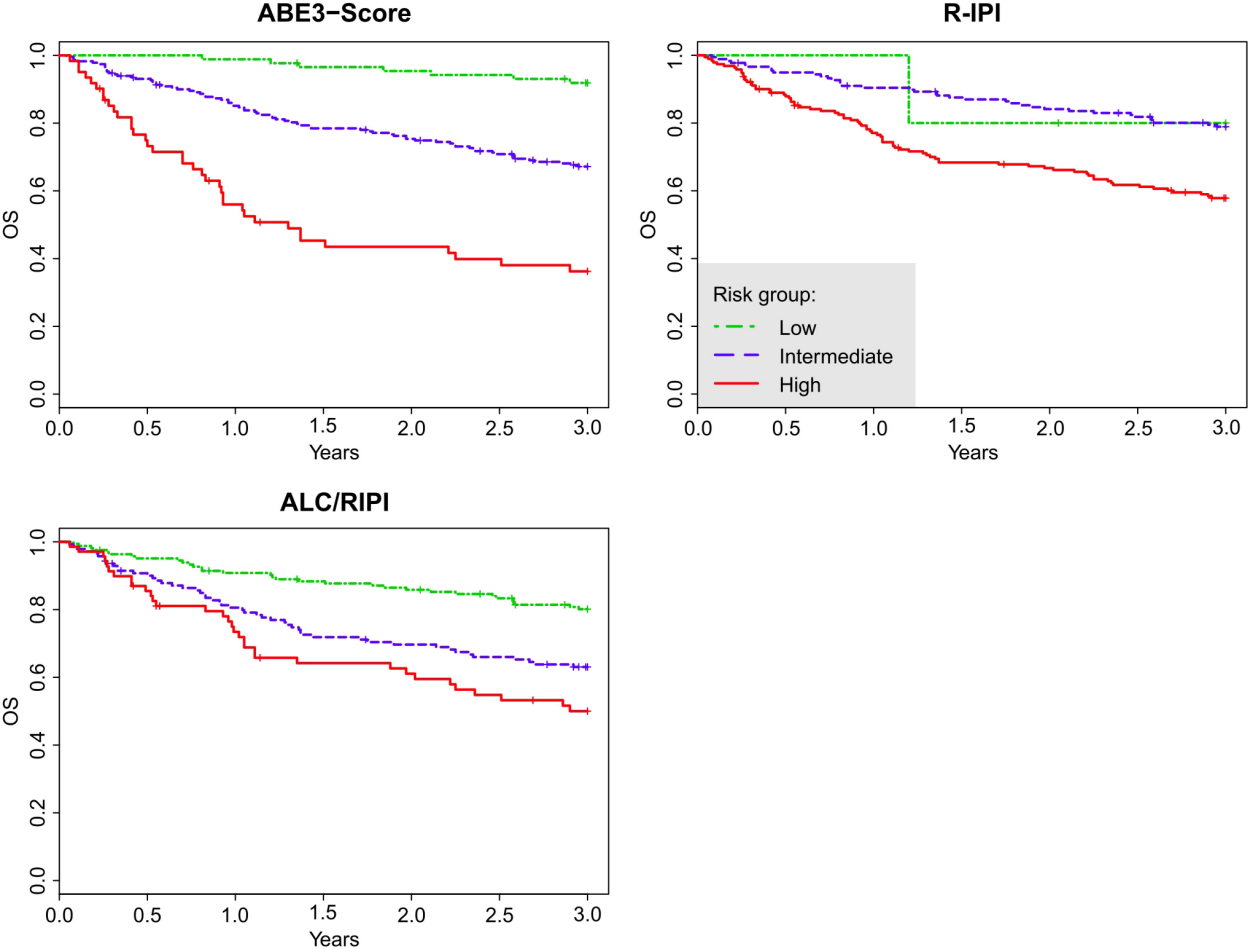
Overall survival (OS) curves for the entire cohort stratified by the 3-level scoring systems: the proposed novel ABE3-Score, and the classical R-IPI, and ALC/RIPI scores.

Let us now compare the ABE4-Score and ABE3-Score systems to existing scoring systems, namely the four-level scores IPI, age-adjusted IPI (AA-IPI), and elderly-IPI (E-IPI), and the three-level scores revised IPI (R-IPI) [6] and its ALC/RIPI form. We fitted a Cox proportional hazards model with each of the scores as the only predictor and calculated the measure of concordance and AIC for each model. Both the ABE4-Score and the ABE3-Score are superior to the existing scoring systems because the ABE4-Score and the ABE3-Score have the highest measures of concordance, which indicate better discrimination. Apart from E-IPI, the ABE4 and ABE3-Scores also have the lowest AIC values in their group which indicate better fit (see the results in Table 6). The estimated 3-year OS by risk groups of individual scoring systems are provided in Table 4 as well as the HR using the lowest risk group as the reference group. These results show better stratification of the risk groups by the ABE4-Score and the ABE3-Score as well. For each of the scoring systems, the estimated OS distribution using the Kaplan-Meier curves are shown in Figures 3 and 4.

**Table 6:** Comparison of the novel scores with the existing ones.

	Concordance (95% CI)	AIC
**Four-level scores**		
ABE4	0.686 (0.637, 0.735)	1304
IPI	0.635 (0.584, 0.686)	1336
Age-adjusted IPI	0.650 (0.599, 0.701)	1325
Elderly IPI	0.665 (0.614, 0.716)	1292
**Three-level scores**		
ABE3	0.676 (0.631, 0.721)	1299
RIPI	0.605 (0.558, 0.652)	1340
ALC/RIPI	0.619 (0.570, 0.668)	1337

*Results from comparison of newly constructed scores (ABE4-Score and ABE3-Score) with several existing scoring systems. The measure of concordance compares the model discrimination, the Akaike Information Criterion (AIC) compares the model fit.*

### Validation

Validation was performed on an independent cohort selected from the same population approximately a year later than collecting the data for the ABE scores construction (see Methods). There is no overlap between the training and validation cohorts. The descriptive statistics of the validation cohort are shown in Table 7. The characteristics of the validation cohort are very similar to the training cohort except for bone marrow involvement, which is notably less present in the validation group (8.8% in the validation group compared to 18.7% in the training group). Also, the median follow-up is significantly lower (3.53 years for the surviving patients) in the validation cohort because it was selected about a year later than the training one. Most of the patients with a long follow-up had already been included in the training group consequently, the validation cohort is biased towards patients with shorter follow-ups. Table 8 compares the hazard rates and the measures of concordance of the ABE3 and ABE4-Score groups in the training and validation cohorts. For ABE4, the validation hazard rates are well within the confidence intervals of the HR in the training cohort. For ABE3, the validation hazard rates are significantly lower. The measure of concordance for ABE4-Score (resp. ABE3-Score) on the validation cohort reads 0.66 (resp. 0.65). Both these values are well within the CI of the respective concordance measures on the training cohort.

**Table 7:** Summary of the prognostic factors in the validation and training cohorts.

	Validation cohort			Training cohort		
Prognostic factor	Min–Max	Median	N (%)	Min–Max	Median	N (%)
Age [years]	60–85	69		60–88	70	
Hemoglobine [g/l]	11–169	128		15–171	126	
No. of extranodal regions	0–4	1		0–5	1	
ECOG score	0–3	1		0–4	1	
Ann Arbor stage	1–4	3		1–4	3	
Sex (male)			76 (46.9)			186 (49.1)
Bulky disease (≥7.5 cm)			58 (35.8)			136 (35.9)
Systemic symptoms present			63 (39.1)			138 (37.1)
Bone marrow affected			14 (8.8)			49 (18.7)
LDH (≥ limit)			98 (62.4)			226 (60.4)
B2M (≥ limit)			68 (54.4)			165 (56.5)

*Comparison of the distribution of the prognostic factors in the validation and the training cohorts.*

**Table 8:** Summary of the validation of the ABE scoring systems.

Score group		HR (95% CI)	P-value	Concordance
**ABE4 in training cohort**				0.686 (0.637, 0.735)
	Low (0)			
	Low-intermediate (I)	4.15 (1.26, 13.66)	0.0191	
	High-intermediate (II)	7.75 (2.42, 24.78)	0.0006	
	High (III)	18.86 (5.78, 61.58)	<0.0001	
**ABE4 in validation cohort**				0.656
	Low (0)			
	Low-intermediate (I)	5.3 (0.68, 41.05)	0.1105	
	High-intermediate (II)	7.18 (0.95, 54.40)	0.0564	
	High (III)	13.36 (1.72, 103.53)	0.0131	
**ABE3 in training cohort**				0.676 (0.631, 0.721)
	Low			
	Intermediate	4.75 (2.19, 10.30)	<0.0001	
	High	13.33 (5.93, 29.95)	<0.0001	
**ABE3 in validation cohort**				0.650
	Low			
	Intermediate	2.12 (0.79, 5.67)	0.1357	
	High	4.81 (1.73, 13.38)	0.0026	

*Results of the univariate 3-year overall survival analysis in the training and the validation data sets: hazard rate (HR) with its 95% CI and P-value based on the Cox regression model. Reference group in all regression models is the lowest risk group. The measure of concordance compares the model discrimination.*

## Discussion

Recent years have brought a lot of information about prognostic role of the absolute lymphocyte count (ALC) and absolute monocyte count (AMC), together with their ratio, LMR. Lymphocytopenia was found to be a strong negative prognostic marker which correlates strongly with the disease burden, patients’ fitness and overall outcome. Negative prognostic roles of low ALC and, inversely, high AMC were explained as results of impaired host-tumor immunosurveillance mechanisms and probably also by the weakening of ADCC activity. Unfortunately, none of these studies used large classes of prognostic factors not included in the conventional IPI score [17], [23], [24]. The present study shows that, if more prognostic factors are included, the role of ALC, AMC, and LMR is overshadowed by different factors.

Diffuse large B-cell lymphoma is a disease of elderly patients, with median age at diagnosis of about 70 years [25]. Despite this fact, most of the predictive scores use the cut-off age of 60 and cover the whole population of DLBCL. Elderly population is markedly different from the younger patients who tend to be in a better physical condition. Consequently, some prognostic factors may have different impact on the overall outcome in the elderly population.

This study attempts to establish the roles of ALC and AMC in an unselected DLBCL population aged over 60, when the role of (at least) all IPI-related factors is taken into account. Analysis of the fourteen clinical and laboratory parameters found only three of them to be sufficient (multivariate) predictors of survival: age ≥70 years, bulk ≥7.5 cm and ECOG ≥1. Surprisingly, ALC, AMC, or LMR were not found to add any predictive power to the multivariate model. Even when tested in the univariate context (each factor as the only predictor of the OS), AMC was found insignificant. The analyses were performed both with continuous values of these variables and with dichotomized values (with the cut-off set to the median of each variable). We suggest that the lack of predictive power of the AMC and ALC can be explained by their close correlation with the bulk and ECOG predictors. These two predictors possibly overshadow the role of AMC and ALC in the final model.

On the other hand, IPI-related factors were found to be strong predictors of OS. First, the cut-off for age was set to the median value of 70 years, in agreement with previously published data [10]. Second, the overall fitness of the patients seems to be more important in the elderly population. In contrast to IPI, patients with only a moderate performance status decrease (ECOG ≥1) showed significantly decreased survival times. Figure 2 shows that the standard dichotomization (ECOG≤1 and ECOG ≥2) does not seem appropriate for the elderly population. Consequently, both the newly proposed ABE scores dichotomize ECOG = 0 and ECOG ≥1. Another important finding is the strong prognostic role of the tumor bulk. This predictor is not included in the IPI score but its relevance has already been confirmed in younger DLBCL patients but not in older population treated with dose-dense regimens [9], [26].

According to the measure of concordance, the four-level ABE4-Score is superior to IPI, AA-IPI, and E-IPI in our dataset. Analogously, the ABE3-Score is superior to both R-IPI and ALC/RIPI. We advise caution when using the measure of concordance to compare a four-level score to a three-level score, however, even in this comparison, the ABE3-Score outperforms all the standard four-level scores (IPI, AA-IPI, and E-IPI). This interpretation is confirmed by the AIC that shows the ABE3-Score to be superior to all other scores except for the E-IPI. However, the stratification of the cohort according to E-IPI lacks the power of the ABE4-Score, because the hazard rates of the E-IPI groups are much lower than the hazard rates of the ABE4 groups. From the practical point of view, both ABE4 and ABE3 scores show the highest span (highest discrimination power) between low- and high-risk groups (59% and 56% difference in OS at 3 years, respectively) compared to all other scores tested. This fact is well captured in the Kaplan-Meier curves (see Figures 3 and 4). IPI, AA-IPI, E-IPI, R-IPI, and ALC/RIPI scores all exhibit some degree of overlapping among the Kaplan-Meier curves for the various risk groups but ABE4 and ABE3 scores show markedly differing outcomes.

It is important to understand the way in which our scores are “fitted” to the data. When fitting a regression model (i.e. tuning its parameters) it comes as no surprise that the fitted model outperforms many other models which were fitted on different datasets. However, in our case, there are no “tunable” parameters that can be fitted to our data. Our training dataset was used only to identify the important predictors and, in case of ECOG, make a decision about their dichotomization. The ABE4-Score was successfully validated on an independent cohort selected from the same population. The score was shown to retain its high discriminatory power and high concordance measure. In case of the ABE3-Score, the validation revealed significantly lower hazard rates in the intermediate and high risk groups. This, together with the simpler interpretation of the ABE4-Score (it represents the number of risk factors present) advocates for the use of the ABE4-Score.

## Conclusions

Prognostic stratification in lymphoma is a “moving target” [16] and our tools should be under continuous revalidation process. Elderly patients are an extremely heterogeneous population and optimal treatment strategy must be adapted with respect to comorbidities and should reflect the true biological age. On the other hand, DLBCL is a curable disease even in the elderly population. Our goal was to postulate a simple, valid and robust prognostic tool for population above the “arbitrary” age limit of sixty years, treated with R-CHOP. We have constructed two variants (three- and four level) of a novel prognostic score. For the routine practice, we recommend the four level ABE4-Score, which is simple to interpret (it represents the number of risk factors present) and robust (it was validated successfully). In conclusion, this study represents the first large analysis of a wide spectrum of prognostic factors in elderly, homogenously treated population with DLBCL. Predictive value of lymphocyte or monocyte count has not been confirmed. The proposed scores based on age, bulk and ECOG were found to be superior to previously published schemes. Other researchers are invited to validate our findings on different populations of elderly patients, homogeneously treated for DLBCL.
